# Entwicklung von Klassifikationskriterien für Uveitiden von der Standardization of Uveitis Nomenclature (SUN) Working Group

**DOI:** 10.1007/s00347-021-01486-2

**Published:** 2021-08-30

**Authors:** Arnd Heiligenhaus, Kai Rothaus, Uwe Pleyer

**Affiliations:** 1 Augenzentrum, St. Franziskus-Hospital, Hohenzollernring 74, 48145 Münster, Deutschland; 2 Universität Duisburg-Essen, Essen, Deutschland; 3 Berliner Institut für Gesundheitsforschung in der Charité (BIH), Campus Virchow Klinikum, Augenklinik, Charité – Universitätsmedizin Berlin, Gliedkörperschaft der Freien Universität Berlin und der Humboldt-Universität zu Berlin, Augustenburger Platz 1, 13353 Berlin, Deutschland

**Keywords:** Uveitis, Klassifikationskriterien, Evidenzbasierte Medizin, Maschinelles Lernen, Forschung, Uveitis, Classification criteria, Evidence based medicine, Machine learning, Research

## Abstract

**Hintergrund:**

Die Standardization of Uveitis Nomenclature (SUN) Working Group ist eine internationale Expertenkommission, die das Ziel verfolgt, eine standardisierte und international anerkannte Terminologie für das Gebiet der Uveitis zu erarbeiten. Dies erscheint angesichts der Forderung nach evidenzbasierter Medizin gerade bei relativ seltenen Erkrankungen wie der Uveitis wichtig.

**Methoden:**

Unter Verwendung formaler Konsensustechniken wurde eine Datenbank von > 4000 Uveitispatienten erstellt, bei denen zuvor eine Mehrheitsübereinstimmung in der Diagnose erzielt wurde. Die Patientendaten wurden innerhalb der Uveitissubklasse analysiert und in einen Trainingssatz und einen Validierungssatz aufgeteilt. Mit maschinellem Lernen (ML) wurden multinomiale logistische Regressionen mit Lasso-Regularisierung auf dem Trainingssatz durchgeführt. Die Genauigkeit der Regeln, die entwickelt wurden, um die Kriterien des maschinellen Lernens auszudrücken, wurde von einem maskierten Beobachter in einer 10 %igen Zufallsstichprobe bewertet.

**Ergebnisse:**

Die Schätzungen der Gesamtgenauigkeit nach Uveitisklassen im Validierungsset waren für alle Uveitisformen hoch: anteriore Uveitiden 96,7 % (95 %-Konfidenzintervall [CI] 92,4–98,6); intermediäre Uveitiden 99,3 % (95 %-CI 96,1–99,9); posteriore Uveitiden 98,0 % (95 %-CI 94,3–99,3); Panuveitiden 94,0 % (95 %-CI 89,0–96,8) und infektiöse posteriore Uveitiden/Panuveitiden 93,3 % (95 %-CI 89,1–96,3).

**Schlussfolgerungen:**

Es werden Klassifikationskriterien präsentiert, die einen hohen Grad an Genauigkeit (geringe Fehlklassifikationsraten) aufweisen und sich daher gut für die künftige klinische und translationale Forschung eignen.

## Abstract (en)

**Background:**

The standardization of uveitis nomenclature (SUN) working group is an international expert committee, which follows the aim to develop a standardized and internationally recognized terminology for the field of uveitis. This appears to be important in view of the demand for evidence-based medicine, especially for relatively rare diseases such as uveitis.

**Methods:**

A databank of > 4000 uveitis patients was compiled using formal consensus techniques, for whom a majority consensus was previously achieved in the diagnosis. The patient data were analyzed within the subclasses of uveitis and divided into a training set and a validation set. Multinomial logistic regressions with LASSO regularization were carried out on the training set with machine learning (ML). The accuracy of the rules that were developed to express the criteria of ML, were assessed by a masked observer in a random sample of 10%.

**Results:**

The estimations of total accuracy according to the uveitis classes in the validation set were high for all forms of uveitis: anterior uveitis 96.7% (95% confidence interval, CI 92.4–98.6%), intermediate uveitis 99.3% (95% CI 96.1–99.9%), posterior uveitis 98.0% (95% CI 94.3–99.3%), panuveitis 94.0% (95% CI 89.0–96.8%) and infectious posterior uveitis/panuveitis 93.3% (95% CI 89.1–96.3%).

**Conclusion:**

Classification criteria are presented, which show a high degree of accuracy (low misclassification rates) and are therefore suitable for future clinical and translational research.

Uveitiden stellen eine heterogene Gruppe von intraokularen Entzündungen dar. Sie werden gruppiert entsprechend dem anatomischen Schwerpunkt der individuellen Augenentzündung (anteriore, intermediäre, posteriore und Panuveitis) und danach, ob sie infektiös, assoziiert mit systemischen autoentzündlichen oder autoimmunen Erkrankungen, auf das Auge beschränkt oder vermutlich immunvermittelt sind.

Die Standardization of Uveitis Nomenclature (SUN) Working Group ist ein internationaler Verbund mit dem Ziel, die Forschung zur Uveitis zu verbessern. Die Gruppe erzielte eine international einheitliche Verwendung der Nomenklatur im klinischen Alltag [1]. Den *etablierten klinisch diagnostischen Kriterien* geht es primär um eine hohe Sensitivität bei der Diagnosestellung. Definitionen und klinisch diagnostische Kriterien der unterschiedlichen Krankheitsbilder werden auch in den vorliegenden Leitlinien von DOG/BVA (Tab. 1) berücksichtigt, die Orientierungshilfen im Sinne von „Handlungs- und Entscheidungskorridoren“ sind. Sie beschreiben, was für eine angemessene Patientenversorgung in der Praxis geboten ist. Entsprechend sind in den darin gelisteten Zielen der augenärztlichen Diagnostik neben dem Nachweis der Entzündungsaktivität die Bestimmung der Lokalisation der Entzündung und Klassifikation, des Schweregrades der Entzündung und der Nachweis von Komplikationen aufgenommen worden.

**Table 1:** 

Leitlinie Nr.	Titel	Stand
14	Uveitis anterior	11/2011
	Interdisziplinäre Leitlinie zur Diagnostik und antientzündlichen Therapie der Uveitis bei juveniler idiopathischer Arthritis. AWMF-Nr. 045/012	02/2021
24a	Uveitis intermedia	06/2020
24b	Nichtinfektiöse Uveitis posterior	08/2017

In den letzten Monaten hat die SUN Working Group eine Reihe von Publikationen vorgelegt, in denen der Prozess der Entwicklung von Klassifikationskriterien für Uveitiden und die resultierende Definition für 25 wichtige Uveitiskrankheitsbilder bekannt gemacht werden. *Klassifikationskriterien* streben nach Spezifität der Diagnosestellung, um in wissenschaftlichen Studien homogene Patientengruppen zu gewährleisten. Sie werden angewendet, um im Einzelfall Erkrankungen für Forschungszwecke zu diagnostizieren. Wie auch die klinisch diagnostischen Kriterien streben Klassifikationskriterien danach, diagnostische Fehleinstufungen zu minimieren.

In dem mehrphasigen Prozess wurden Uveitiskrankheitsbilder aufgearbeitet und klassifiziert. Mit einem zuvor etablierten standardisierten Vokabular und Dimensionsset wurden von Experten in vorläufigen Datensätzen die Diagnosekriterien herausgearbeitet. Für jedes einzelne Krankheitsbild wurden zunächst Leitbefunde abgestimmt, auf ihre Validität geprüft, mittels Machine Learning ein Klassifikator trainiert und die Kriterien und Ausschlusskriterien der unterschiedlichen Krankheitsbilder zusammengetragen.

Machine Learning (maschinelles Lernen; ML) ist ein Ansatz aus dem Bereich der künstlichen Intelligenz. Beim maschinellen Lernen wird Wissen aus Erfahrung gewonnen, indem anhand gegebener Beispiele (Trainingsdaten) Modelle trainiert werden und somit eine vorgegebene Aufgabe erlernt wird. Wesentlich bei dem ML-Prozess ist die Abstraktionsfähigkeit des Systems, also die Eigenschaft, auch auf der Basis unbekannter Daten adäquate Ergebnisse zu produzieren. Mittels neuer Beispiele (Validierungsdaten) wird geprüft, ob lediglich die Trainingsdaten auswendig gelernt wurden oder ob das Verfahren in der Lage ist, auf diese neuen Fälle zu verallgemeinern, also die zugrunde liegenden Gesetzmäßigkeiten erlernt wurden.

Nachfolgend wird ein orientierender Querschnitt der in den Klassifikationsprozess aufgenommenen Krankheitsbilder dargestellt.

## Material und Methoden

In einem ersten Schritt wurden ein standardisiertes Vokabular und ein Set von Dimensionen zur Beschreibung der Uveitiskrankheitsbilder erarbeitet. Auf dieser Grundlage wurden von 76 teilnehmenden Untersuchern für den weiteren Prozess 5766 Datensätze von Patienten mit jeweils einem von 25 klinisch relevanten Uveitiskrankheitsbildern (jeweils ca. 150 bis 250 Patienten) retrospektiv zusammengetragen sowie für die einzelnen Krankheitsbilder sehr typische Bilddokumentationen (z. B. Fundusbilder, Fluoreszeinangiographien und OCT-Bilder). Die Fallsammlungen der 25 Gruppen wurde durch ein Komitee überprüft und abgestimmt (inklusive Konsensuskonferenzen, nominale Gruppentechnik, anonyme Abstimmung, >75 % Übereinstimmung).

**Figure 1:**
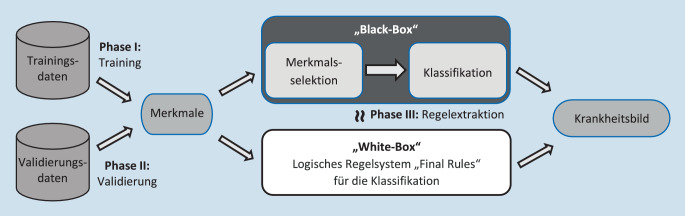

Danach folgten Phasen mit maschinellem Lernen (Abb. 1). Für jede anatomische Uveitisklasse wurde separat ein eigenes Modell trainiert (Phase I) und das erlernte Modell validiert (Phase II). Zusätzlich wurde das Modell mittels der Trainingsdaten in ein einfaches logisches Regelsystem transferiert (Phase III) und anschließend auf 10 % der Daten gegen die Bewertung eines maskierten Experten validiert.

Nach einer geeigneten Rekodierung der Daten wurde in der ersten Phase das Modell trainiert. Zunächst wurden für die einzelnen Krankheitsbilder die entscheidenden Merkmale ausgewählt (Verfahren von Boruta), sodass eine Balance zwischen Sparsamkeit (so wenig Merkmale wie nötig) und Genauigkeit gewährleistet wurde. Mit der reduzierten Merkmalsmenge wurde ein Klassifikationsverfahren trainiert, sodass die Übereinstimmung mit dem zutreffenden Uveitiskrankheitsbild maximiert wurde. Es wurden unterschiedliche ML-Algorithmen verglichen, und schlussendlich wurde das Verfahren der multinomialen logistischen Regression gewählt. Das trainierte Modell wurde in einer zweiten Phase validiert. Dabei wurden bislang nicht verwendete Datensätze mithilfe des trainierten Modells verarbeitet, um anhand der erreichten Genauigkeit die Abstraktionsfähigkeit des Modells zu bewerten. Das Modell kapselt das erlernte Wissen in einer „Black-Box“, und der Entscheidungsweg ist somit für den Menschen nur bedingt interpretierbar. Aus diesem Grund wurde in einer dritten Phase ein für den Arzt gut zu handhabendes logisches Regelsystem („Final Rules“) abgeleitet, welches auf den Trainings- und Validierungsdaten stets dieselbe Entscheidung trifft wie das abstrakte Modell, das in Phase I trainiert wurde. Die Güte dieses Regelsystems wurde bewertet durch den Vergleich der Beurteilung eines zusätzlichen, maskierten Readers mit den „Final Rules“ und der Konsensdiagnose.

## Ergebnisse

In den nunmehr vorliegenden Publikationen der SUN Working Group [2–27] werden die Klassifikationskriterien von 25 klinisch relevanten Krankheitsbildern zusammengefasst (Tab. 2). Exemplarisch werden nachfolgend 3 in der Praxis häufige und klinisch besonders relevante Krankheitsbilder dargestellt.

**Table 2:** 

Akute posteriore multifokale plakoide Pigmentepitheliopathie (APMPPE)
Akutes retinales Nekrosesyndrom
Behçet-Erkrankung mit Uveitis
Birdshot-Chorioretinitis
Fuchs-Uveitis-Syndrom
Herpes-simplex-Virus(HSV)-anteriore Uveitis
Intermediäre Uveitis, Nicht-Pars-planitis-Typ
Juvenile idiopathische Arthritis(JIA)-assoziierte Uveitis
Multifokale Choroiditis mit Panuveitis
„Multiple evanescent white dot syndrome“ (MEWDS)
Multiple Sklerose-assoziierte intermediäre Uveitis
Pars planitis
„Punctate inner choroiditis“ (PIC)
Sarkoidose-assoziierte Uveitis
Serpiginöse Choroiditis
Spondyloarthritis(SpA)/HLA-B27-assoziierte anteriore Uveitis
Sympathische Ophthalmie
Syphilitische Uveitis
Toxoplasmose-Retinitis
Tuberkulöse Uveitis
Tubulointestinale Nephritis mit Uveitis (TINU)
Varicella-Zoster-Virus(VZV)-anteriore Uveitis
Vogt-Koyanagi-Harada-Erkrankung
Zytomegalievirus(CMV)-Retinitis

### Spondyloarthritis/HLA-B27-assoziierte anteriore Uveitis

Spondyloarthritis (SpA) umfasst ein Spektrum von entzündlichen Gelenkerkrankungen, zu dem die ankylosierende Arthritis, reaktive Arthritis, Psoriasisarthritis und Arthritis bei entzündlichen Darmerkrankungen zählt. Die Prävalenz der SpA liegt unter 2 %. Eine häufige Begleiterkrankung ist die Uveitis (10–25 %, entsprechend der SpA-Form), die typischerweise HLA-B27 assoziiert ist und einen rezidivierenden akuten, einseitigen oder beidseitig alternierenden Verlauf nimmt, wobei bis zu 15 % später in einen chronischen Verlauf übergehen.

Von den in die Studie [7] aufgenommenen 251 Patienten mit SpA/HLA-B27-assoziierter anteriorer Uveitis wurden 184 Patienten mit diagnostischer Mehrheitsübereinstimmung in die maschinelle Lernphase aufgenommen. Diese ausgewählte Gruppe wurde mit anderen Gruppen von anteriorer Uveitis (CMV, HSV, VZV, Fuchs-Uveitis-Syndrom, JIA, TINU, Sarkoidose und Syphilis) hinsichtlich demografischer und uveitisbezogener Parameter verglichen. Patienten mit akutem und rezidivierend akutem Verlauf wurden anderen mit chronischem Verlauf gegenübergestellt. Die nach anschließender Auswertung mit maschinellem Lernen resultierenden Klassifikationskriterien werden in Tab. 3 zusammengefasst. Die Gesamtgenauigkeitsschätzung für anteriore Uveitiden betrug 97,5 % (95 %-Konfidenzintervall [CI] 96,3–98,4) im Trainingsset und 96,7 % (95 %-CI 92,4–98,6) im Validierungsset. Die Fehleinstufungsrate für SpA/HLA-B27-assoziierte anteriore Uveitis betrug im Trainingsset 0 % und im Validierungsset 3,6 %.

**Table 3:** 

*Kriterien*	
1.	Nachweis einer anterioren Uveitis
	a. Vorderkammerzellen
	b. Wenn vordere Glaskörperzellen vorliegen, ist deren Schwere geringer als der Vorderkammerentzündung
UND entweder (sowohl #2 und #3) ODER #4	
2.	Charakteristischer Uveitisverlauf
	a. Akuter oder rezidivierend akuter, unilateraler oder wechselseitiger Verlauf ODER
	b. Chronischer Verlauf mit Anamnese von rezidivierenden akuten Schüben, unilateral oder wechselseitiger Verlauf mit Entwicklung eines chronischen Verlaufes
UND	
3.	ASAS-definierte Spondyloarthritis (axial oder peripher) und/oder HLA-B27-positiv
ODER	
1.	Chronische Uveitis mit sowohl ASAS-definierter Spondyloarthritis (axial oder peripher) UND HLA-B27-positiv
*Ausschlusskriterien*	
1.	Positive Syphilisserologie mit *Treponema-pallidum*-Test
2.	Nachweis einer Sarkoidose (entweder bilaterale hiläre Adenopathie in der Thoraxröntgenaufnahme oder Gewebebiopsie mit dem Nachweis nicht verkäsender Granulome)
3.	Kammerwasserprobe PCR-positiv für Zytomegalievirus, Herpes-simplex-Virus oder Varicella-Zoster-Virus

*ASAS* Assessment of SpondyloArthritis international Society, *PCR* Polymerasekettenreaktion

### Fuchs-Uveitis-Syndrom

Das Fuchs-Uveitis-Syndrom (FUS) nimmt im typischen Fall einen nahezu asymptomatischen schleichenden Verlauf. Die Patienten bemerken das Krankheitsbild oft erst durch etwaige Langzeitfolgen, zu denen insbesondere Glaskörpertrübungen und Kataraktbildung zählen. Die Diagnose wird häufig erst bei einer augenärztlichen Routinekontrolle gestellt. Typische klinische Zeichen umfassen einen milden bis moderaten Vorderkammerzellbefund, charakteristische punkt- und sternförmige Keratopräzipitate (meist der gesamten Hornhautrückfläche), eine Irisatrophie – die häufig zu einer Heterochromie führt – sowie einen milden Zellbefund im vorderen Glaskörper. Synechien treten typischerweise nicht auf. Weitere typische Langzeitfolgen sind die okuläre Hypertension und das Glaukom. Kontrovers diskutiert wird aktuell, ob sich hinter dem morphologischen Bild eines FUS eine heterogene Gruppe unterschiedlicher, z. T. infektiöser Erkrankungen verbirgt.

Von den in die Studie [27] aufgenommenen 249 FUS-Patienten wurden 146 Patienten mit diagnostischer Mehrheitsübereinstimmung in die maschinelle Lernphase aufgenommen. Diese FUS-Gruppe wurde mit anderen Gruppen von anteriorer Uveitis (CMV, HSV, VZV, SpA/HLA-B27, JIA, TINU, Sarkoidose und Syphilis) hinsichtlich demografischer und uveitisbezogener Parameter verglichen. Die nach anschließender Auswertung mit maschinellem Lernen resultierenden Klassifikationskriterien werden in Tab. 4 zusammengefasst. Die Gesamtgenauigkeitsschätzung für anteriore Uveitiden betrug 97,5 % im Trainingsset und 96,7 % (95 %-CI 92,4–98,6) im Validierungsset. Die Fehleinstufungsrate für FUS betrug im Trainingsset 4,7 % und im Validierungsset 5,5 %.

**Table 4:** 

*Kriterien*	
1.	Nachweis einer anterioren Uveitis
	a. Vorderkammerzellen
	b. Wenn Glaskörperzellen vorliegen, sollte auch eine Vorderkammerentzündung vorliegen
	c. Kein Hinweis für aktive Retinitis
UND	
2.	Unilaterale Uveitis
UND	
3.	Anzeichen eines Fuchs-Uveitis-Syndroms
	a. Heterochromie ODER
	b. Unilaterale diffuse Irisatrophie UND stellataförmige Keratopräzipitate
UND	
4.	Weder Endotheliitis noch noduläre, münzförmige endotheliale Läsionen
*Ausschlusskriterien*	
1.	Positive Syphilisserologie mit *Treponema-pallidum*-Test
2.	Nachweis einer Sarkoidose (entweder bilaterale hiläre Adenopathie in der Thoraxröntgenaufnahme oder Gewebebiopsie mit dem Nachweis nicht verkäsender Granulome)
3.	Kammerwasserprobe PCR-positiv für Zytomegalievirus, Herpes-simplex-Virus oder Varicella-Zoster-Virus

*PCR* Polymerasekettenreaktion

### Akutes retinales Nekrosesyndrom

Das akute retinale Nekrose(ARN)-Syndrom ist ein sehr seltenes Krankheitsbild (Inzidenz in United Kingdom 0,5–0,63/1 Mio. Einwohner/Jahr), das durch Viren der Herpesgruppe (vorrangig HSV‑1 und -2, seltener VZV) initiiert wird und mit einer schweren Begleitentzündung einhergeht. Einige Patienten weisen eine zurückliegende oder gleichzeitige Herpeserkrankung des zentralen Nervensystems (Meningitis oder Enzephalitis) auf. Es wird diskutiert, dass genetische Risikofaktoren in der Immunantwort an der Entstehung des Krankheitsbildes beteiligt sein könnten. Die prompte Initiierung und prolongierte Gabe antiviraler Medikamente kann den Krankheitsverlauf bessern und das Risiko der Erkrankung am anderen Auge reduzieren. Dennoch ist Visusprognose oftmals schlecht, was oft der späten Diagnosestellung und dem verzögerten Therapiebeginn geschuldet ist.

Von den in die Studie [22] aufgenommenen 252 Patienten mit ARN wurden 186 Patienten mit diagnostischer Mehrheitsübereinstimmung in die maschinelle Lernphase aufgenommen. Diese ausgewählte Gruppe wurde mit anderen Gruppen von infektiöser posteriorer Uveitis/Panuveitis (CMV, Toxoplasmose, Syphilis, Tuberkulose) hinsichtlich demografischer und uveitisbezogener Parameter verglichen. Die nach anschließender Auswertung mit maschinellem Lernen resultierenden Klassifikationskriterien werden in Tab. 5 zusammengefasst. Die Gesamtgenauigkeitsschätzung für infektiöse posteriore Uveitiden/Panuveitiden betrug 92,1 % im Trainingsset und 93,3 % (95 %-CI 88,2–96,3) im Validierungsset. Die Fehleinstufungsrate für ARN betrug im Trainingsset 15 % und im Validierungsset 11,5 %. Die meisten Fehleinstufungen gab es gegenüber CMV- und Toxoplasmose-Retinitis.

**Table 5:** 

*Kriterien*	
1.	Nekrotisierende Retinitis unter Einbeziehung der peripheren Netzhaut
UND (entweder #2 ODER #3)	
2.	Nachweis einer Infektion mit entweder HSV oder VZV
	a. Positive PCR für entweder HSV oder VZV entweder aus Kammerwasser- oder Glaskörperproben
ODER	
3.	Charakteristisches klinisches Bild
	a. Umlaufende oder konfluierende Retinitis UND
	b. Retinale Gefäßeinscheidung und/oder -okklusionen UND
	c. Mehr als minimale Vitritis
*Ausschlusskriterien*	
1.	Positive Syphilisserologie mit *Treponema-pallidum*-Test
2.	Intraokulare Probe PCR-positiv für Zytomegalievirus oder *Toxoplasma gondii* (es sei denn, dass eine Immunschwäche vorliegt, morphologischer Nachweis für >1 Infektion, das charakteristische klinische Bild einer akuten retinalen Nekrose und die intraokulare Probe PCR-positiv für entweder HSV oder VZV)

*PCR* Polymerasekettenreaktion

## Schlussfolgerungen

Zusammenfassend ist es der SUN Working Group gelungen, unter Zuhilfenahme formaler Konsensustechniken und künstlicher Intelligenz relevante Klassifikationskriterien der klinisch relevanten Uveitisformen zu erarbeiten, die den Rahmen für künftige klinische und translationale Studien vorgeben werden. Bedeutungsvoll ist, dass einige für die klinische Betreuung von Uveitispatienten sehr relevante Aspekte hier nicht mit eingeflossen sind.
